# The Effect of Pre-sowing Seed Treatment with Metal Nanoparticles on the Formation of the Defensive Reaction of Wheat Seedlings Infected with the Eyespot Causal Agent

**DOI:** 10.1186/s11671-016-1305-0

**Published:** 2016-02-16

**Authors:** Olga Panyuta, Viktoriya Belava, Svitlana Fomaidi, Olena Kalinichenko, Mykola Volkogon, Nataliya Taran

**Affiliations:** Educational and Scientific Centre “Institute of Biology”, Taras Shevchenko National University of Kyiv, 64, Vladymyrska str., Kyiv, 01601 Ukraine

**Keywords:** Metal nanoparticles, Lipid peroxidation, Lectins, Wheat, *Pseudocercosporella herpotrichoides*, *Oculimacula yallundae*, 82.39.-k, 82.70.Dd

## Abstract

The paper presents research data of lipid peroxidation and lectin activity in wheat seedlings at seed treatment with solution of metal nanoparticles (Zn, Ag, Fe, Mn, Cu) and sole solution of copper nanoparticles under the high pathogen infection background of *Pseudocercosporella herpotrichoides* (Fron) Deighton (synonym: *Oculimacula yallundae* (Wallwork & Sponer) Crous & W. Gams). It was shown that investigated nonionic colloidal solutions of biogenic metals have the antioxidant effect through the inhibition of the synthesis of lipid peroxidation products. The increase of lectin activity levels during the early plants ontogenesis stages was observed in wheat seedlings infected with pathogen pre-treated with the mixture of metal nanoparticles.

## Background

Nowadays, nanopreparations are gaining more and more interest in various areas of our life. They are used as biological stimulants and micronutrients as they have specific advantages over traditional solutions: they do not break up by heat and light, ensure complete plant surface moistening, can be fully absorbed by plants, and remain on the surface under the rain. Moreover, working solutions of nanoparticles stay active at long-term storage.

The interaction of nanoparticles and biological objects occur on the cellular level, enhancing the efficiency of biochemical processes in plants. Nanoparticles take part in maintenance of microelements balance, which indicates their high biological activity. Obtained nanoforms of copper, zinc, and iron, unlike their salts, are less toxic. Thus, copper nanoparticles are seven times less toxic as compared to sulfate salt of copper. Zinc nanoparticles are 30 times and iron nanoparticles are 40 times less toxic as compared to their corresponding sulfate salts [1].

The use of metal nanoparticles in agriculture for pre-sowing seed treatment and/or for the foliar application improves the quality of seeding material, increases plants resistance to pathogens, and enhances overall crops productivity. Furthermore, metal nanoparticles can be used in organic production [2, 3].

In context of the abovementioned, the aim of our research was to investigate the effect of nonionic colloidal solutions of nanoparticles of biogenic metals on the formation of defensive reactions of winter wheat seedlings on pathogen infection of *Pseudocercosporella herpotrichoides* (Fron) Deighton (synonym: *Oculimacula yallundae* (Wallwork & Sponer) Crous & W. Gams) [4].

## Methods

Wheat plants of Myronivska 808 variety which are susceptible to the *P. herpotrichoides* infection were grown in greenhouse conditions on sand and treated with a solution of nonionic metal nanoparticles (zinc, silver, iron, manganese, copper) and sole nonionic solution of copper nanoparticles.

Mixtures of metal nanoparticles were obtained from the Department of Construction Materials Technology and Material Sciences of National University of Life and Environmental Sciences of Ukraine. Colloidal solutions were received by dispersing zinc, silver, iron, manganese, and copper granules in water by electric pulses with an amplitude of 100–2000 A [5].

Pre-sowing wheat seed treatment was performed with the working solution of metal nanoparticles in ratio 1:100 (colloidal solution to water, respectively) for 4 h. Then, seeds were washed with distilled water and placed into the incubator at 25 °C for 24 h. Seeds in control variant were treated only with the distilled water. In 24 h, seeds were sown in the chemically neutral containers (8 × 8 × 9 cm) and infected with the conidia suspension of *P. herpotrichoides* pathogenic fungus. High virulent strain 543 7/1 of *P. herpotrichoides* (Fron) Deighton (current name *O. yallundae* (Wallwork & Sponer) Crous & W. Gams) was obtained from the Laboratory of Crops Immunity to Diseases of Institute of Plant Protection UAAS) [6].

The intensity of lipid peroxidation (LPO) was evaluated by measuring the contents of malonic dialdehyde (MDA) using 2-thiobarbituric acid reaction [7]. Light absorption was recorded at *λ* = 532 nm.

Extraction of soluble proteins and determination of the lectin activity (LA) were performed by agglutination reaction at the lowest protein concentration that have caused the agglutination of rat erythrocytes [8]. Lectins activity was estimated by formula as an inverse value to the minimum concentration of the protein that have caused the erythrocyte agglutination reaction and was represented in (mg/ml)^−1^. The total protein content was determined using the Bradford assay [9].

The reliability differences between the variants were assessed by Student’s *t* test at *p* ≤ 0.05 significance level [10].

## Results and Discussion

The general plant response to abiotic and biotic stresses, including pathogens, is the increase of reactive oxygen species (ROS) levels, which stimulate lipid peroxidation and are accompanied by disorders in structural and functional integrity of cell membranes. Thiobarbituric acid reactive substances (TBARS), including MDA, which are accumulated in plant cells under the stress conditions as the result of lipid peroxidation are used as the LPO markers. Malonic dialdehyde is highly active low-molecular hydrophilic compound, which is normally present in plant cells at low concentrations. Since MDA is one of the most stable products of lipid peroxidation, its content in plant tissues is widely used to assess the activity of LPO process. Accumulation of lipid peroxidation products can be used for evaluation of plant resistance to the stressors action of various nature [11].

**Fig. 1:**
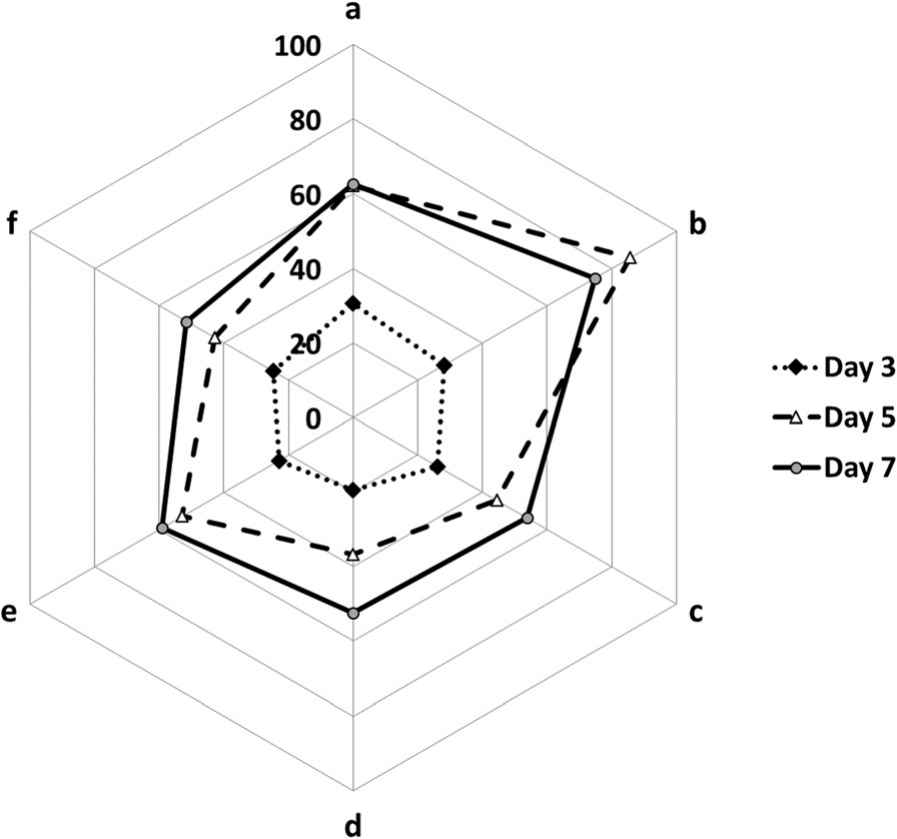
TBARS content (nmol/g FW) in wheat seedlings at pre-sowing seed treatment with metal nanoparticles solutions and subsequent infection of plants with the suspension of *P. herpotrichoides* conidia. *a* control, *b* seed infection with *P. herpotrichoides*, *c* seed treatment with copper nanoparticles solution, *d* seed treatment with copper nanoparticles solution and subsequent seed infection with *P. herpotrichoides*, *e* seed treatment with metal nanoparticles (zinc, silver, iron, manganese, and copper) solution, *f* seed treatment with metal nanoparticles (zinc, silver, iron, manganese, and copper) solution and subsequent seed infection with *P. herpotrichoides*

The study of accumulation dynamics of lipid peroxidation products in winter wheat plants at pre-sowing seed treatment with biogenic metal nanoparticles solutions had resulted in the reduction of TBARS in seedling tissues as compared to the untreated plants in control (Fig. 1). Under the use of metal nanoparticles mixture, the TBARS content was lower comparing to control by 5.5–24.9 %. Seed treatment with copper nanoparticles solution had led to the TBARS content decrease comparing to control by 14.1–28.7 %.

Plants infection with the suspension of conidia of *P. herpotrichoides* fungus had increased the content of TBARS in seedlings obtained from non-treated seeds comparing to the non-infected control or any other experimental variant. The maximum content of lipid peroxidation products (almost 1.4 times higher than in control) in these seedlings was observed on the fifth day after infection, which indicates the development of plant stress state caused by pathogenesis. The lowest TBARS content in infected plants was observed in the plants which seeds were treated with the copper nanoparticles solution on the fifth day after infection, which was almost 1.7 times lower than in control. Taking into account the fungicidal properties of copper, we assume that copper nanoparticles, like silver [12, 13], interact with fungus structures and interrupt normal course of physiological reactions, affect internal homeostasis, trigger the pathogen death, and thus prevent the development of pathological process, which is reflected in the reduction of TBARS content.

It should be noted that the investigated solutions of nonionic nanoparticles can act as antioxidants [14], inhibiting formation of TBARS, namely malonic dialdehyde, that led to lower comparing to control level of TBARS in non-infected seedlings as well as in infected seedlings treated with metal nanoparticles.

Pathogen induces the synthesis of pathogenesis-related (PR) proteins in plants, which are involved in the formation of the plant immune response. It was established that PR proteins are always present in plants in residual amounts. Stress, caused by pathogen infection, considerably increases the concentration and activity of these proteins. Lectins are protective proteins with low molecular weight. They are capable of binding to chitin and belong to the PR-4 family. By interacting with the surface of bacterial cells, spores, and fungi hyphae, they cause cessation of pathogens growth and prevent development of pathological process. The significant increase of the LA level is occurred under the influence of pathogens and action of various preparations aimed to improve plants resistance to the pathogens [15].

Study of lectin activity in winter wheat plants under the pathogens infection (root rot, septoria disease, harmful bacteria) indicates the changes of LA value, which correlates with the development of other disease signs. The maximum LA was observed at the fourth to ninth day after infection [16, 17, 15]. These data clearly indicate the direct participation of lectins in the formation of protective reactions of plants against pathogens.

Churilov [18] has demonstrated that ultra disperse metal powder had stimulated growth and development of medicinal plants. In addition, usage of metal nanopowders, comparing to controls, had increased the content of biologically active substances like ascorbic acid, carotene, and carbohydrates in these plants. Thus, it can be assumed that pre-sowing seed treatment with nonionic solutions of biogenic metal nanoparticles will influence the formation of plant defense reactions under the action of either abiotic or biotic stressor.

**Fig. 2:**
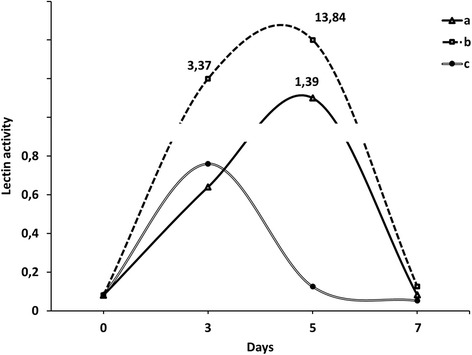
Dynamics of lectin activity ((mg/ml)^**−1**^) in leaves of wheat seedlings in variants with seed treatment with the solutions of metal nanoparticles. *a* control, *b* seed treatment with copper nanoparticles solution, *c* seed treatment with metals nanoparticles solution (zinc, silver, iron, manganese, and copper)

The study of LA dynamics in the tissues of above ground parts of seedlings from the control variant have shown gradual increase of LA index to the fifth day with subsequent decrease to the initial levels on the seventh day (Fig. 2).

Pre-sowing treatment of winter wheat seeds with metal nanoparticles mixture had led to a minor increase (in 1.2 times) of LA relatively to control values and values which were obtained on the third day, followed by a gradual decrease. Pre-sowing treatment of winter wheat seeds with copper nanoparticles solution has induced a significant increase (in 5.3 times) of LA level compared to the control value on the third day with its subsequent increase in four times compared to the values obtained on the fifth day. Observations have shown that on the fifth day, the LA value which was obtained in this variant was almost ten times higher than in control. Such fluctuations of LA values might have been caused by pre-sowing treatment of winter wheat seeds with solutions of biogenic metals nanoparticles, similar to action of xenobiotics that activate defensive reactions in plants.

**Fig. 3:**
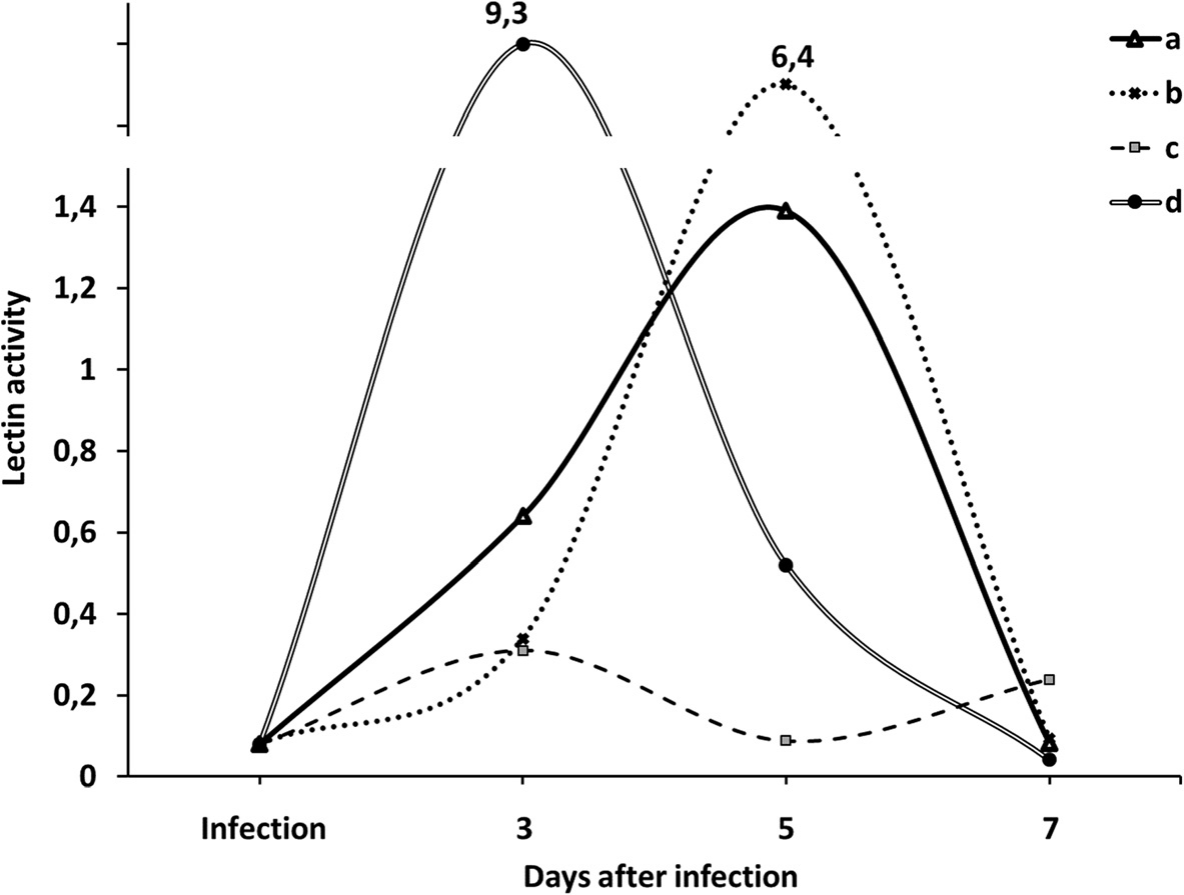
Dynamics of lectin activity ((mg/ml)^**-1**^) in leaves of wheat seedlings in variants with seed treatment with the solutions of metal nanoparticles and the subsequent infection with the suspension of *P. herpotrichoides* conidia. *a* control, *b* seed infection with *P. herpotrichoides*, *c* seed treatment with copper nanoparticles solution and subsequent seed infection with *P. herpotrichoides*, *d* seed treatment with metal nanoparticle solution (zinc, silver, iron, manganese, and copper) and subsequent seed infection with *P. herpotrichoides*

Induction of lectin activity in non-treated variants was observed on the fifth day, same as in control, but with much higher level (in 4.6 times), which was caused by the activation of defense reactions due to the pathogenesis (Fig. 3).

In seedlings obtained from seeds which were treated with metal nanoparticles mixture and subsequently infected with the suspension of *P. herpotrichoides* conidia, the maximum LA level (14.6 times higher than in control) was observed on the third day after infection. Thus, pre-sowing treatment of winter wheat seeds with the mixture of metal nanoparticles under the pathogen infection conditions has increased the activity of defensive proteins, lectins, notably higher at early stages, hence indicating the role of metal nanoparticles as exogenous inducers of endogenous defense reactions. However, the lowest level of lectin activity comparing to other variants was observed in seedlings obtained from variants with seed treatment with copper nanoparticles solution and subsequent plants infection with *P. herpotrichoides* fungus. It should be noted that in this case, LA on the fifth day was minimal (in 3.5 times lower than on the third day and in 2.7 times lower than on the seventh). Overall, at pre-sowing treatment of winter wheat seeds with copper nanoparticles and pathogen stress conditions, the lowest level of lectin activity was observed throughout the experiment period. At the same time, as it also should be noted, seedlings in these variants had possessed the highest relative growth of aboveground parts.

The results obtained in the presented study of the formation of wheat defensive reactions at pathogenesis by changing the activity of protective proteins—lectins are consistent with the formation of defensive reactions observed at the level of accumulation of lipid peroxidation products.

It was shown that levels of lectin activity on the seventh day in all variants have decreased. The inverse correlation between the changes of total protein content and activity of protective proteins, lectins, was observed in all the studied variants.

## Conclusions

At wheat infection with *P. herpotrichoides* (Fron) Deighton (synonym: *O. yallundae* (Wallwork & Sponer) Crous & W. Gams) pathogen, which causes the oxidative burst in the cells, the studied nonionic solutions of biogenic metals nanoparticles acted as an antioxidants, inhibiting the formation of thiobarbituric acid reactive substances of lipid peroxidation, namely malonic dialdehyde. Based on the results, it can be assumed that metal nanoparticles may increase antioxidant properties of cells under phytopathogen stress condition and improve physiological condition of plants.

The nature of overall dynamics of lectin activity in the control and infected plants was the same, but the maximum values of lectin activity were substantially higher under the stress conditions, indicating induction of lectin activity by pathogen. Pre-sowing treatment of winter wheat seeds with nonionic solution of metal nanoparticles (zinc, silver, iron, manganese, and copper) caused the increase of LA, even more evident in the infected plants. Same trend was also observed in the non-treated variants, which might indicate the induction of endogenous defense reactions to pathogen infection in wheat seedlings. Winter wheat seedlings obtained from the seeds treated with solution of copper nanoparticles demonstrated similar dynamics of lectin activity to that in control, but five to ten times higher than LA values in control. However, in variants with plant infection, the level of lectin activity was the lowest, due to the fungicide properties of copper.
